# Principles of Treatment in Placental Presentations

**Published:** 1847-08

**Authors:** J. Y. Simpson

**Affiliations:** Professor of Midwifery in the University of Edinburgh


*Principles of Treatment in Placental Presentations.* By J. Y. Simpson, M. D., Professor of Midwifery in the University of Edinburgh.—In a late very interesting and very able paper, on unavoidable hæmorrhage, published in the Lancet for March 27th, I observed that the author, Mr. Barnes, argues on the idea that I recommended the complete separation and detachment of the placenta before the child, as a general rule of practice in all cases of placental presentation. Many other members of the profession appear to have taken up the same impression. I have always, however, maintained a very different doctrine. From the first observations which I published on the subject, up to the present time, I have upheld that the practice of detaching the placenta before the child, in unavoidable hæmorrhage, was a method to be had recourse to in cases where the other recognized modes of management were insufficient, or unsafe, or altogether impossible of application; and I have always looked upon this new method as possessing especial value, from its thus presenting to us a rational means of treatment in precisely those more formidable varieties of this obstetric complication, in which all former plans of practice were attended with extreme hazard or extreme difficulty.

As I am anxious to avoid future error and misconception on this head, I would beg leave here to take the liberty of enumerating briefly, and without entering into special details, the different general principles of treatment which, in my humble opinion, ought to guide our practice in this important and anxious class of cases.

Setting aside, then, those minor and palliative measures for moderating the attendant flooding that are generally adopted by practitioners in all cases of uterine hæmorrhage, where time permits of their employment, (such as quietude, the supine position, cold, &c.,) I hold that our management of placental presentations, when either labour or such severe flooding as demands interference does at last supervene, should be regulated on the following principles:—

I. *In some cases no active interference is required.*—In placental presentations, we deem ourselves called upon to interfere operatively with the avowed object and purpose of saving the patient from the dangers of the attendant hæmorrhage. Hence it necessarily follows that it would not be requisite to adopt any special form of artificial aid or delivery, if, in any case or cases, this complication were accompanied with little or no flooding. Now, in some instances of partial presentation of the placenta, the flooding ceases altogether, or abates to a safe degree, when, during the natural progress of labour, the membranes rupture and the head descends. And in some rare cases of complete presentation of the placenta, where the vascular bleeding structure of the placental mass has become obstructed and obliterated previously to the supervention of labour, little or no hæmorrhage has accompanied the process of delivery. In other instances, before any operative aid can be applied, the hæmorrhage suddenly and entirely ceases in consequence of the placenta becoming totally separated, and expelled by the advancing head of the infant. Under such circumstances, and others, where the present or prospective dangers attendant upon operative interference would be evidently greater than the present or prospective dangers attendant upon the existing degree of hæmorrhage, any form of forced delivery would, I believe, be improper. But cases of placental presentation in which we can thus leave the delivery altogether to Nature are rare. Generally, we require to adopt some active measures, with the special object of saving the patient from the actual or threatened dangers of the hæmorrhage. These measures should, I conceive, be one or other of the plans which I have now to proceed to mention—viz., 1, the artificial evacuation of the liquor amnii; or 2, the artificial extraction of the child; or 3, the artificial separation of the placenta.

II. *Artificial Evacuation of the Liquor Amnii.*—In partial presentations of the placenta, rupturing the exposed portion of membranes (according to those principles that are generally followed in accidental floodings) is a measure which sometimes proves quite adequate to arrest or abate the hæmorrhage to such an extent, that the delivery may be afterwards entirely committed to the effects of Nature. Various old authors, as Daventer, Deleurye, and Astrue, have described this same plan of treatment as applicable to complete, as well as to partial presentations of the placenta, with this difference, that in the complete variety, the liquor amnii is evacuated, not by puncturing the membranes only, but by perforating the opposing placental structure with a trocar, catheter, or other analogous instrument. And the later records of midwifery contain several cases in which this perforation of the placenta has, in complete presentations of the organ, been successfully adopted, both as regards the mother and the infant.

Several high authorities, however, in midwifery, have altogether repudiated the evacuation of the liquor amnii, both in partial and in complete placental presentations. They have done so principally under the idea, that if this measure failed to suppress the flooding, the previous escape of the waters would render any subsequent practice that might be required more difficult of execution. This objection certainly applies to turning, as a subsequent practice, but it does not apply to artificial detachment of the placenta as an ulterior measure of treatment.

The artificial evacuation of the liquor amnii, by perforating either the placenta or membranes, affords assuredly a simple, but by no means a certain, method of restraining the flooding in placenta prævia. It is a practice which is undoubtedly attended with less success in unavoidable than in accidental hæmorrhage. But still I believe it to be a mode of treatment, to which we may occasionally have recourse with great advantage, especially if there is originally a large quantity of liquor amnii present, and if the flooding is great, while the os uteri is still small and undilatable. We must beware, however, of trusting too much or too long to this, or to any mere palliative measures. Whatever we do, should, if possible, be always done before the hæmorrhage is allowed to proceed to such an extent as to induce any very marked symptoms of constitutional debility and depression in our patient. If a decided state of exhaustion has been allowed to supervene, either of the two remaining and ulterior measures—extraction of the child, or extraction of the placenta—will be but too liable to prove futile and unsuccessful in their results.

III. *Artificial Extraction of the Child.*—This forms the general principle of management upon which unavoidable hæmorrhage has hitherto been treated by most authors and practitioners. The professed object of the practice is this: by forcing the delivery of the child, and thus emptying the uterus, the organ is thrown into full contraction, and hence further loss of blood prevented. The mode in which the indication is fulfilled is in some degree regulated by the state of advancement of the infant, its presentation, &c. In a great proportion of cases the accompanying hæmorrhage requires interference at so early a stage of the labour, that the only proper and possible mode of delivery is by the operation of turning; and various authors, as Drs. Denman, Burns, Hamilton, Conquest, and others, speak of turning as the *sole* and *only* mode of treatment applicable to cases of placental presentation. The great objection to it is the imminent danger which the mother necessarily runs, from the risk of some laceration of the cervix uteri during this mode of forcible delivery; and any degree of laceration of this part is especially dangerous in placental presentations; for in placenta prævia the structure of the cervix is extremely vascular, being permeated with those numerous and enlarged vessels which are always developed, in a high degree, in the uterine walls opposite the seat of the placenta. The laceration of these vessels leads to immediate danger, from draining hæmorrhage after delivery, and to more remote danger, from inflammation being liable to spring up in the torn and wounded sinuses of this part, and extreme uterine phlebitis following as a direct consequence. But still I hold turning to be the proper mode of practice in unavoidable hæmorrhages which cannot be restrained by less active measures, and where immediate delivery is demanded, with the os uteri well dilated, or easily dilatable, and the child still alive, or presenting transversely.

Besides turning, other modes of artificial delivery of the infant are occasionally resorted to in placental presentations. If the attendant flooding is such as not to require forced delivery till after the waters are evacuated, and the head well advanced in the passages, then version would be dangerous and inapplicable, and the use of the forceps offers the safest and easiest mode of extracting the infant. Further: in original pelvic presentations extraction may be at any time effected, when required, by seizing and dragging at the feet of the child.

V. *Artificial Separation of the Placenta.*—The arrestment of unavoidable flooding by total detachment of the placenta should, I believe, be our line of practice when the combination of circumstances is as follows—viz., the hæmorrhage is so great as to show the necessity of interference, and is not restrainable or restrained by milder measures, (such as the evacuation of the liquor amnii;) but, at the same time, turning, or any other mode of immediate and forcible delivery of the child, is especially hazardous or impracticable, in consequence of the undilated or undeveloped state of the os uteri, the contraction of the pelvic passages, &c. Or, again, the death, prematurity, or non-viability of the infant, may not require us to adopt modes of delivery, for its sake, that are accompanied (as turning is) with much peril to the mother, provided we have a simpler and safer means, such as the detachment of the placenta, for at once commanding and restraining the hæmorrhage, and guarding the life of the parent against the dangers of its continuance. Hence, as I have elsewhere stated, I believe that the suppression of the flooding by the total detachment of the placenta will be found the proper line of practice in severe cases of unavoidable hæmorrhage, complicated with an os uteri so insufficiently dilated and undilatable as not to allow of version being performed with perfect safety to the mother: therefore, in most primiparæ; in many cases in which placental presentations are (as very often happens) connected with premature labour and imperfect development of the cervix and os uteri: in labours supervening earlier than the seventh month; when the uterus is too contracted to allow of turning; when the pelvis or passages of the mother are organically contracted; when the child is dead; when it is premature, and not viable; and where the mother is in such an extreme state of exhaustion as to be unable, without immediate peril of life, to be submit ed to the shock and dangers of turning, or forcible delivery of the infant. This enumeration is far from comprehending all the forms of placental presentations that are met with in practice; but it certainly includes a considerable proportion of the cases of this obstetric complication, and among them, all, or almost all, of the most dangerous and most difficult varieties of unavoidable hæmorrhage. In adopting the practice, one error, which I would strongly protest against, has been committed in some instances. Besides completely detaching and extracting the placenta, the child has subsequently been extracted by direct operative interference. If the hæmorrhage ceases, as it usually does, upon the placenta being completely separated, the expulsion of the child should be subsequently left to Nature, unless it present preternaturally, or the labour afterwards show any kind of complication, which of itself would require operative interference under any other circumstances. Both to detach the placenta and extract the child would be hazarding a double instead of a single operation.

*Comparative Mortality attendant upon Turning, and upon the total Separation of the Placenta.*—One circumstance which strongly led me to advocate, in unavoidable hæmorrhage, the preference of the detachment of the placenta to the operation of turning the child, was the fact of the great mortality which followed the latter operation, as contrasted with the few mothers that died when the placenta was spontaneously expelled, or accidentally extracted before the infant. In speaking of the relative maternal mortality resulting from the two modes of practice, Mr. Barnes very properly points out, that when I spoke of the mortality attendant upon the separation of the placenta before the child as amounting to one in fourteen only, (ten in 141 mothers having died,) I had included cases in which the placenta was thrown off spontaneously before the child, along with others in which it was artificially detached; and he doubts if the results would not be "widely different" if the statistics comprehended the latter class of cases only, "in which the severe operation of detaching the placenta, by the introduction of the hand, had been resorted to." The best answer to this objection consists in a statement of the results hitherto obtained from the practice of artificially detaching the placenta.

"Seventeen cases," says Dr. West, "have been recorded in the English journals, during the past fifteen months, of detachment of the placenta before the birth of the child in cases of placenta prævia. In the case recorded by Dr. Simpson, to whom it had been communicated by Mr. Cripps, the placenta was removed by an ignorant midwife, and ten hours elapsed before the child was born, during which time, however, no hæmorrhage took place. In sixteen out of the seventeen cases, the bleeding is said to have ceased immediately on the detachment of the placenta; but Dr. Everitt mentions, that although the flooding abated on the separation of the placenta, it did not entirely cease until after the application of cold externally; and he insists on the fact as proving, in cases of this kind, the hæmorrhage comes from the uterine as well as the placental ends of the lacerated veins. The life of the mother was preserved in every case but one, (out of the seventeen,) and then the previous hæmorrhage had been so profuse as almost to exhaust the patient, who died three hours after delivery. All the children were still-born, except in the case related by Mr. Stickings."

I do not stop to inquire whether in one and all of these seventeen cases the artificial detachment and extraction of the placenta ought to have been followed. At present I adduce them, not as affording evidence of the propriety of the practice, but as affording evidence of its safety.

In proof of the maternal mortality under the old and recognised forms of practice being greatly higher than under the proposed plan of the extraction of the placenta before the child, Mr. Barnes refers, apparently with some hesitation, to the statistics collected by Dr. Churchill and myself, as showing that one in every three mothers was usually lost in placental presentations. Among 174 cases of unavoidable hæmorrhage collected by Dr. Churchill, forty-eight mothers died. I have now before me a carefully collected list of 654 cases of placental presentations reported by Mauriceau, Portal, Giffard, Smellie, Rigby, Clarke, and Collins, Schweighauser, Lachapelle, Drs. John and Francis Ramsbotham, Lee, Lever, and Wilson. Among these 654 cases, 180 mothers died, or one in three-sixteenths. In corroboration of the correctness of the statistical view which Dr. Churchill and I have taken of the extent of maternal mortality in unavoidable hæmorrhage, I would further beg to refer Mr. Barnes to the observations of Dr. Robert Lee. In his Midwifery Lectures, (pp. 370, 371,) published in 1844, Dr. Lee states a number of statistical facts regarding uterine hæmorrhage from placental presentations, and, amongst other matters, he mentions the result to the mothers in a considerable number of cases. I shall throw all his evidence on this last point into a tabular form.

*Maternal Mortality in Seventy-two Cases of Placental Presentations noted by Dr. Lee.*

| Reporters. | No. of cases reported. | No. of mothers lost. |
|---|---|---|
| Dr. Clarke | 14 | 1 |
| Dr. Collins | 11 | 2 |
| Dr. Ramsbotham | 19 | 8 |
| Dr. Lee, | 38 | 14 |
| | 72 | 25 |

Hence, according to Dr. Lee's collection of statistics, the maternal mortality in unavoidable hæmorrhage, amounting to twenty-five in seventy-two cases, is rather more than one in three. And this evidence of Dr. Lee will probably be regarded as the stronger, seeing that it is totally unprejudiced in its character; for in 1845, Dr. Lee called into doubt the accuracy of all collections of statistical data made by others, and which led to the idea, that the general maternal mortality in unavoidable hæmorrhage was so great, as to approach one in three. At that time he was, I believe, unaware of the general result of his own previously published collection of statistical data relative to the point in question.—*Lancet.*

